# Changing paradigm of malnutrition among Bangladeshi women of reproductive age and gaps in national Nutrition Policies and Action Plans to tackle the emerging challenge

**DOI:** 10.3389/fpubh.2024.1341418

**Published:** 2024-10-16

**Authors:** Shusmita Khan, M. Moinuddin Haider, Kanta Jamil, Karar Zunaid Ahsan, Saiqa Siraj, Afrin Iqbal, Gustavo Angeles

**Affiliations:** ^1^Data for Impact, Chapel Hill, NC, United States; ^2^University of North Carolina at Chapel Hill, Chapel Hill, NC, United States; ^3^Health Systems and Population Studies Division (HSPSD), icddr,b, Dhaka, Bangladesh; ^4^Independent Consultant, Melbourne, VIC, Australia; ^5^Nutrition International, Dhaka, Bangladesh; ^6^Maternal and Child Health Division, International Centre for Diarrhoeal Disease Research (ICDDR), icddr,b, Dhaka, Bangladesh

**Keywords:** nutrition, malnutrition, obesity, maternal health, double burden, Bangladesh, policy

## Abstract

**Objective:**

The main objective of this paper is to document the changing paradigm of malnutrition in Bangladesh and estimating how this is creating an intergenerational risk. This paper also examines national policy responses to tackle the silent epidemic of double burden of malnutrition.

**Methods:**

Publicly available datasets of five Bangladesh Demographic and Health Surveys were used to see the changing paradigm of malnutrition among Bangladesh women. In addition to that, four national policies concerning, maternal and child health; and nutrition were reviewed using CDC’s 2013 Policy Analytical Framework.

**Results:**

In Bangladesh, the share of ever-married women aged 15–49 who were underweight declined sharply between 2007 and 2017–2018, from 30 to 12%. In the same period, the proportion of women who were overweight or obese increased from 12 to 32%. Despite remarkable progress in reducing undernourishment among women, the share of well-nourished remained unchanged: 58% in 2007 and 56% in 2017–2018, mainly due to the shift in the dominant burden from undernutrition to overnutrition. This shift occurred around 2012–2013. Currently, in Bangladesh 0.8 million of births occur to overweight women and 0.5 million births occur to underweight women. If the current trend in malnutrition continues, pregnancies/births among overweight women will increase. Bangladesh’s existing relevant policies concerning maternal health and nutrition are inadequate and mostly address the underweight spectrum of malnutrition.

**Discussion:**

Both forms of malnutrition pose a risk for maternal and child health. Underweight mothers are at risk of having anemia, antepartum/postpartum hemorrhage, and premature rupture of membranes. Maternal obesity increases the risk of perinatal complications, such as gestational diabetes, gestational hypertension, and cesarean deliveries. Currently, around 24% of the children are born to overweight/obese mothers and 15% to underweight mothers. Bangladesh should revise its national policies to address the double burden of malnutrition among women of reproductive age across pre-conception, pregnancy, and post-natal stages to ensure optimum maternal and child health.

## 1 Introduction

As per the World Health Organization (WHO)—malnutrition encompasses a combination of both overnutrition or undernutrition leading to a transformation in body composition, outcome, and/or reduced body function (1). The term malnutrition covers three comprehensive groups of conditions: undernutrition (*wasting, stunting, and underweight*); overnutrition (*overweight and obesity*); and micronutrient-related malnutrition (2).

On the other hand, the double/dual burden of malnutrition (DBM) is characterized by the coexistence of both undernutrition and overweight/obesity in the same population. This can be within particular/respective individuals, households, and communities and can spread across the life-course (3, 4). Generally, in any context, the DBM becomes distinct in any population when reduction in one form of malnutrition becomes slower than the progression of the other form. This means that in any population, the DBM will be more apparent when the reduction rate of undernutrition is slower than the increasing rate of overnutrition (5). This occurs when a country experiences a nutrition transition because of demographic and socioeconomic changes (6).

Due to the complicated combination and contrasting levels of nutrients, understanding the spectrum of malnutrition and its double burden, along with its effects on human health, requires a holistic conceptualization. Thus, tackling all forms of malnutrition has become one of the largest global-health challenges in today’s world (7). The transition to a more mechanized lifestyle influenced by economic and income growth, and the shift toward processed and genetically modified diets, are resulting into a fast-evolving and more complex nutrition paradigm, worldwide (8).

Despite much improvements in many human development indexes, nearly one in three people around the world is living with at least one type of malnutrition (1). In 2014, almost 462 million adults globally were underweight, and another 1.9 billion were overweight or obese (9). One might assume that a major proportion of this dual burden would be in developed countries; however, the reality is that the major proportion of this double/dual burden is among people living in developing countries in Asia and Africa (10, 11). In India, national level data show that the DBM is quite evident (12). Moreover, studies document that almost 50% of urban Indian adults are overweight or obese (13, 14). Similar evidences are available for other developing countries, such as Egypt, China, Mexico, Philippines, and South Africa.

A recent paper analyzing data from 55 LMICs revealed that between 1990 and 2018, while the prevalence of underweight came down in 35 countries, at the same time, prevalence of overweight mounted in 50 countries (15). The same paper projected that by the year 2030, more than 50% of women of reproductive age in 22 LMICs is likely to be overweight or obese, and 24 LMICs were projected to experience the DBM. Bangladesh was one of the countries projected to experience the DBM among women aged 15–49 years. A review paper published in 2013 similarly indicated that the country would experience the shift from underweight to overweight in around 2014 (16). Many recent studies (17–23) also indicated on coexistence of various forms of DBM in Bangladesh.

The economic, developmental, health, and social impacts of DBM are serious and has lasting effects on individuals, their families, communities, and the nation as a whole. Furthermore, for women of reproductive age, the risk is greater because these women give birth to children whose health and nutrition largely depend on the health and nutrition of their mothers. Therefore, having malnourished women of reproductive age poses a great risk of having malnourished future generation (24–26).

This paper focuses on the shift from undernutrition to overnutrition, and the co-existence of DBM among Bangladeshi women aged 15–49 years. The paper also estimates the number of children born to malnourished mothers who are likely to have health risks. Lastly, the paper examines Bangladesh’s policy responses to tackle this silent epidemic of DBM.

## 2 Materials and methods

### 2.1 Data

This paper analyzed data from five nationally representative, Bangladesh Demographic and Health Surveys (BDHS) (27) conducted in 2004, 2007, 2011, 2014 and 2017–18 (28–31). Details on the sample of analysis used in this study, including sample sizes, and inclusion and exclusion criteria of the unit of analysis (women) are presented in the Supplementary Table S1. The BDHSs apply a standardized technique of multistage cluster sampling and the questionnaires are comparable across years. This paper used publicly available anonymous data from the DHS program dataset site (32).

To estimate the number of ever-married women of reproductive age (ages 15–19 years) and the number of annual live births, we used World Population Prospects 2022 (33), the BDHS 2017–18 and World Population Prospects 2019. Details on the calculations are presented in the Supplementary Tables S2, S3.

### 2.2 Definitions and measurement approaches

We considered the global cut-off of body mass index (BMI), to define underweight (BMI < 18.5 kg/m^2^) and overweight (BMI ≥ 25.0 kg/m^2^) women (WHO, 2010a). The BMI is a function of weight in kilograms (kg) by the square of height in meters (m^2^) Since there is a separate BMI Cut-off for the Asian population and Bangladesh falls under that, the BMI cut-off for the Asian population – defining underweight (BMI < 18.5 kg/m^2^) and overweight (BMI ≥ 23.0 kg/m^2^) of women (86, 8).

The analysis included non-pregnant and non-lactating ever-married women of reproductive age (EMWRA; age 15–49 years). For bivariate analysis, age was categorized in two groups (15–29 years and 30–49 years) because more than 80% of children are born to women age less than 30 years (31). We analyzed women’s nutritional status by place of residence (rural and urban) and household wealth quintiles (poor: the bottom two quintiles; middle: third quintile; and rich: the upper two quintiles).

### 2.3 Policies analyzed

To understand the national policy situation, the authors analyzed the following four relevant national guiding documents.

- Bangladesh National Strategy for Maternal Health 2019–2030 (34).
- National Nutrition Policy 2015 (35).
- Second National Plan of Action for Nutrition 2016–2025 (36).
- Bangladesh National Nutrition Council’s (BNNC) Addressing Bottlenecks for the Coverage of Nutrition Sensitive Interventions in Bangladesh (37).

Nutrition is a complex issue that requires multisectoral policy responses. However, because this paper focuses on identifying the extent of DBM on women age 15–49 years and how it is engendering intergenerational challenges, national policies directly linked with maternal and child health and nutrition were reviewed and analyzed.

### 2.4 Policy analysis framework

**Figure 1:**
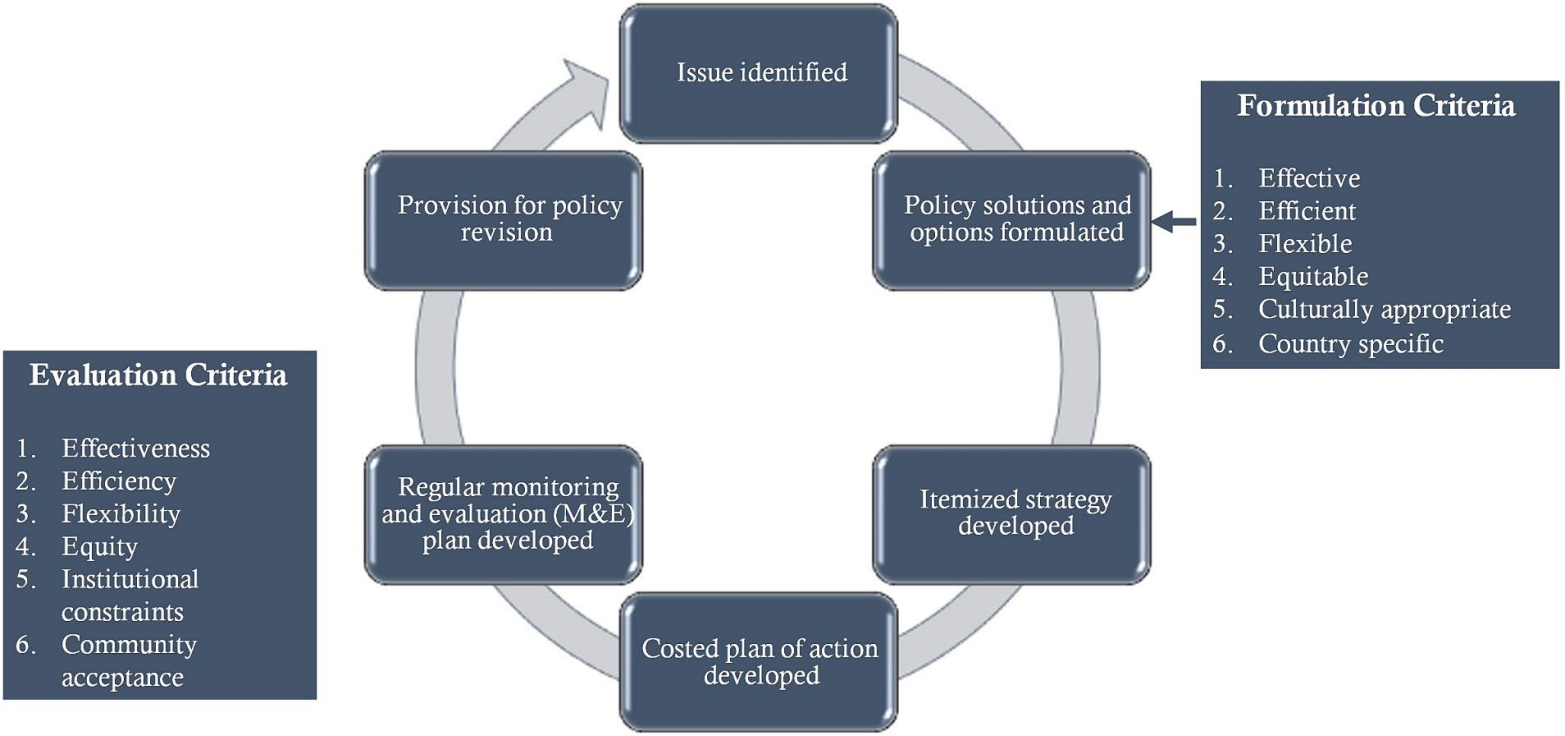
Steps to analyze national policy documents, adapted from CDC’s 2013 policy analytical framework.

To analyze the national documents, the authors used and adapted the U.S. Centers for Disease Control and Prevention’s (CDC) 2013 Policy Analytical Framework (38) (see Figure 1). This framework provides a roadmap for analyzing national policy responses.

To identify policy solutions and options in the documents analyzed, a set of framing questions were developed (Supplementary Table S4). A scoring process (Supplementary Table S5) was also prepared to determine policy effectiveness (steps 3–6 in the framework).

## 3 Results

### 3.1 Prevalence of malnutrition among women in Bangladesh

#### 3.1.1 Status, trends, and projections

**Figure 2:**
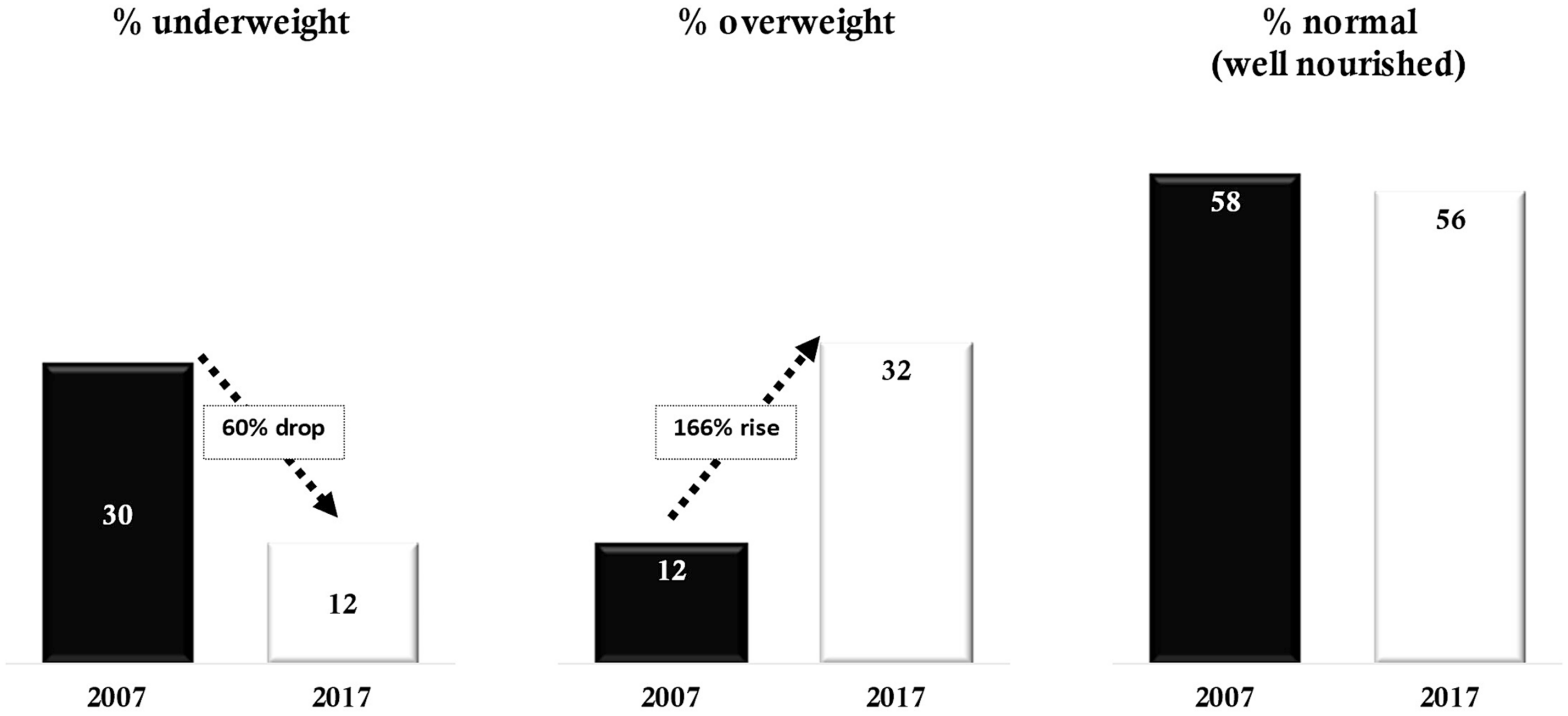
Nutritional status of women of reproductive age, 2007 and 2017–18 BDHS.

Data from the 2007 and 2017–2018 BDHSs show that within just 10 years, the share of underweight women of reproductive age declined from 30 to 12%, representing a 60% drop. On the other hand, the portion of overweight/obese women of reproductive age increased at a higher rate, from 12% in 2007 to 32% in 2017–18, representing a 166% increase in this same period. Despite significant improvement in reducing undernutrition among women, the share of well-nourished women (BMI = 18.5–24.9 kg/m^2^) remained almost unchanged at 58% in 2007 and 56% in 2017 (Figure 2).

**Figure 3:**
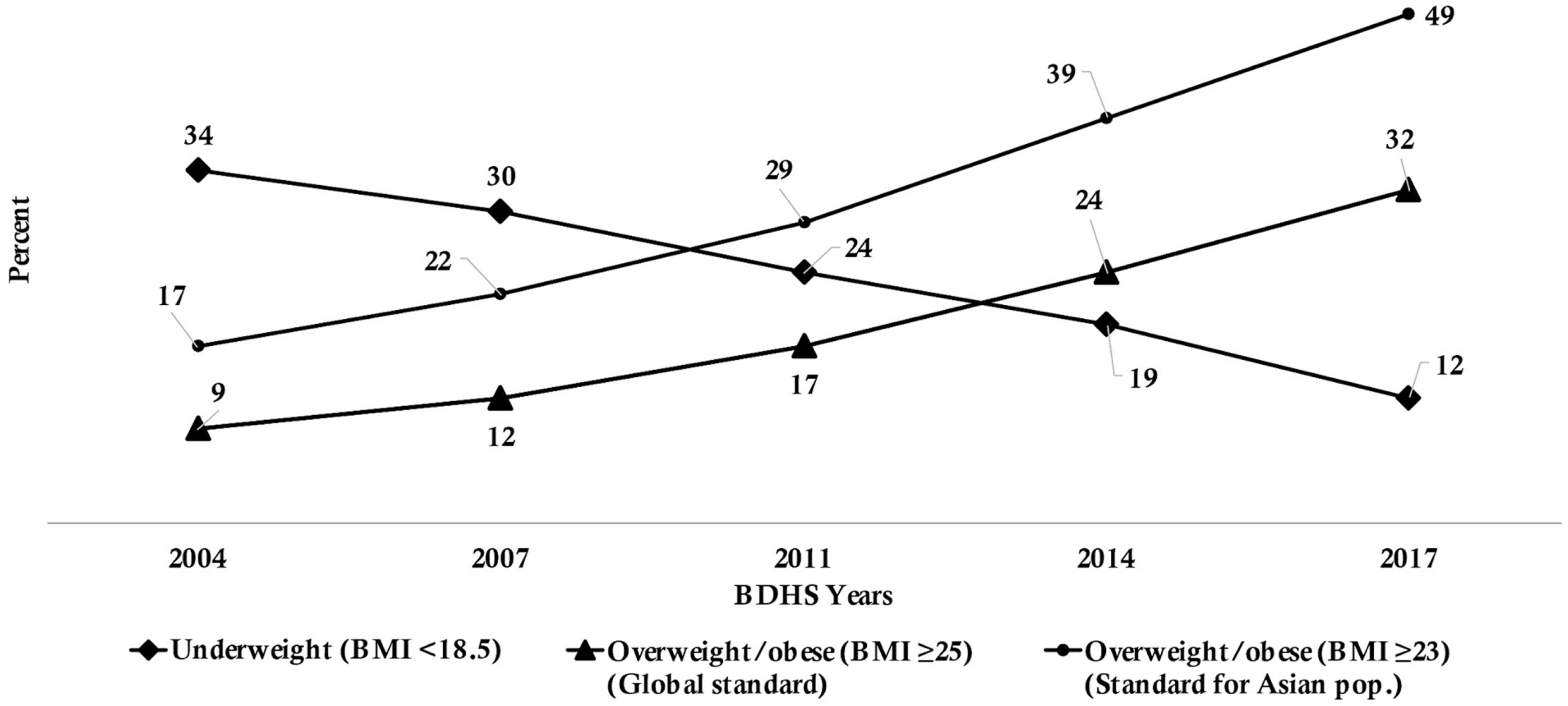
Percentage of underweight and overweight/obesity among women of reproductive age, 2004 to 2017–18, Bangladesh.

According to the BDHSs, the mean BMI of women age 15–49 years increased from 20.2 in 2004 to 23.3 in 2017–18. In 2004, almost 34% of Bangladeshi women age 15–49 years were suffering from underweight. This dropped to 30% in 2007, 24% in 2011, 19% in 2014, and 12% in 2017–18. However, among the same population, the prevalence of overweight increased from 9% in 2004 to 32% in 2017–18. This rise becomes sharper if it is measured using the BMI cut-off for the Asian population, with 17% in 2004 to 49% in 2017–18. This change over of the dominant burden from underweight to overweight occurred around 2012–2013 (BMI ≥25 kg/m^2^) or around 2009–2010 (BMI ≥23 kg/m^2^; Figure 3).

#### 3.1.2 The extent of the burden

**Figure 4:**
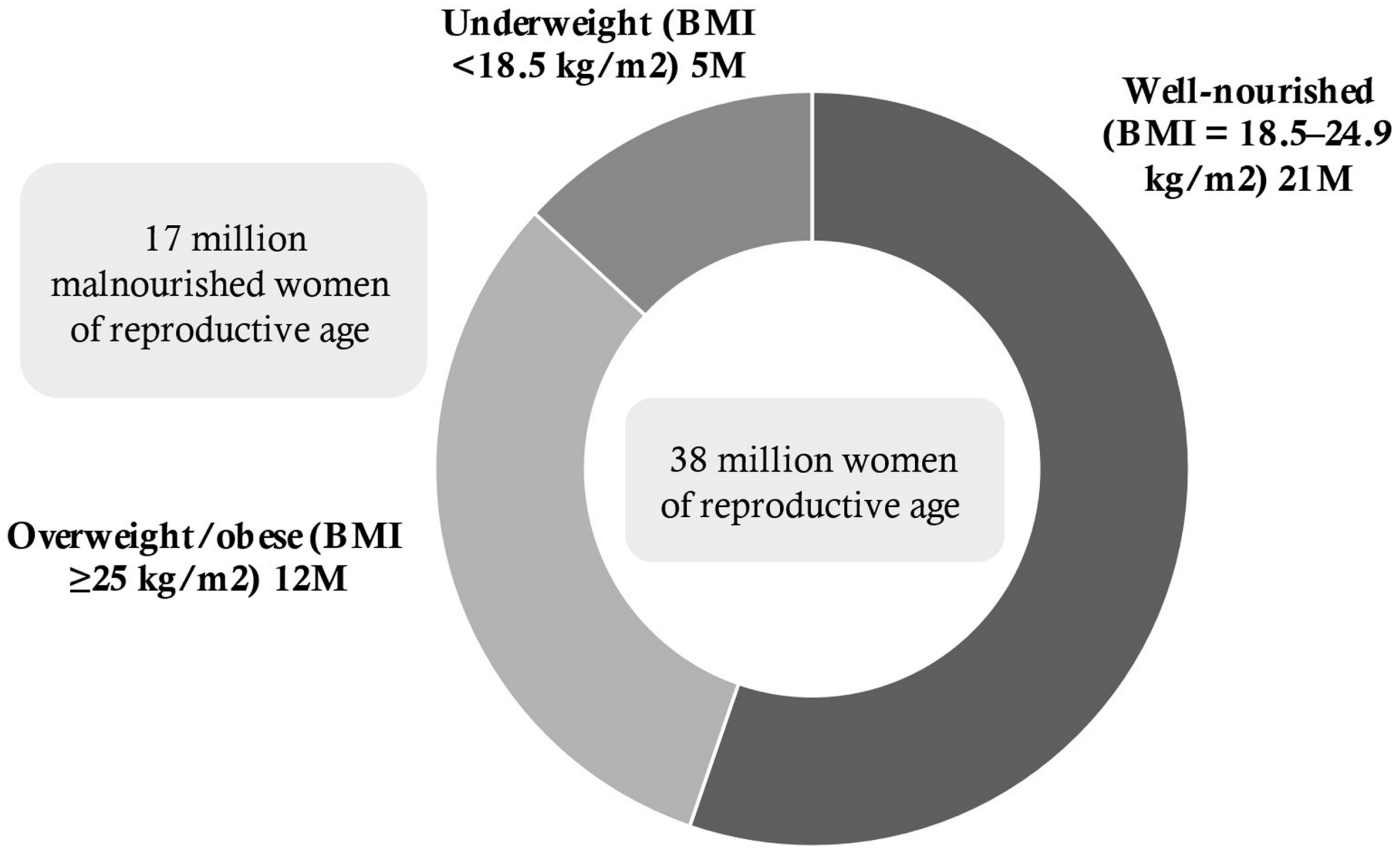
Estimated number (in millions) of malnourished women of reproductive age, 2017–18, Bangladesh.

There were around 38 million EMWRA in Bangladesh in 2022 (33). Factoring in the BDHS 2017–18 prevalence of overweight/obesity among EMWRA, it is estimated 17 million of these women were malnourished. Of these 17 million women, 5 million were underweight and 12 million were overweight/obese (Figure 4). With 3.4 million annual births, it is estimated that a total 1.3 million children were born to malnourished mothers (0.5 million to underweight EMWRA and 0.8 million to overweight/obese EMWRA). Detailed calculations can be found in Supplementary Table S3.

Younger women and women living in rural areas and are from poorer households are more like to be underweight. In contrast, older women, those living in urban areas, and women in more affluent households and more likely to be overweight. However, over time the proportion of underweight have fallen, and overweight levels have increased across age, place of residence and all economic groups (Table 1).

**Table 1:** Percentage of underweight and overweight among ever-married women (15–49 years) by background characteristics, 2007 to 2017–18, Bangladesh.

Background characteristics	Underweight (%; BMI < 18.5 kg/m^2^)		Overweight (%; BMI ≥ 25 kg/m^2^)	
	2007	2017–18	2007	2017–18
Age				
Ages 15–29	31	15	8	24
Ages 30–49	28	10	15	39
Place of Residence				
Urban	20	9	29	43
Rural	33	13	8	28
Household Economic Status				
Poor (Bottom two wealth quintiles)	39	18	4	19
Middle (Third wealth quintile)	33	11	7	31
Rich (Upper two wealth quintiles)	19	7	22	45

### 3.2 National policy responses

Based on the policy analysis, it was found that existing policies regarding maternal health and nutrition were not adequate and principally addressed the underweight aspect of malnutrition. The Bangladesh National Strategy for Maternal Health 2019–2030, a thorough official strategy, is a critical document for maternal health in Bangladesh. This policy has eight (8) targets, focusing on fertility/family planning, antenatal care, delivery, postnatal care, maternal mortality, and new born mortality rates. Even if the strategy recognizes “overweight and obesity” as concerns, under the “persisting gap,” it does not offer any certain action item for solving the concern. Rather for women age 15–49 years, the strategy fundamentally nuclei on the effects of being underweight, with nothing on the expanding proportion of pregnancies and births among overweight/obese women.

In terms of policies focusing on nutrition, the National Nutrition Policy 2015 – a government gazette – serves as the key nutrition-related government document. This policy document likewise largely focuses on undernutrition, regardless of recognizing “reducing maternal overweight (BMI >23)” as an indicator for accomplishing optimal nutrition for all.

The third most important national document for nutrition is the Second National Plan of Action for Nutrition 2016–2025, which aims “the first 1,000 days, adolescent girls, pregnant and lactating women, older populations, physical, mental, and cognitive disabled.” Although the document is intended to cover the whole population, it does not specify any activities for non-pregnant and non-lactating women or cover women across their reproductive age and predominantly aims on “maternal underweight.” Though the plan of action outlines some activities for the “prevention and control of obesity and non-communicable diseases,” it does not specify the target groups intended for those specific activities.

The fourth document analyzed for this paper was “Addressing Bottlenecks for the Coverage of Nutrition Sensitive Interventions in Bangladesh,” published in 2021 by the BNNC. This document successfully identifies the bottlenecks and ways forward for improving coverage of nutrition-sensitive interventions in Bangladesh, keeping the decreasing trend of underweight and increasing trend of overweight in mind. However, because it is not a policy/strategy document, it may prove difficult for the agencies working toward a well-nourished Bangladesh to implement the suggested follow-up activities.

Table 2 presents the analysis of the documents based on effectiveness, efficiency, flexibility, and equity.

**Table 2:** Status of the national policy/strategy/plan of action analyzed.

Criteria	Bangladesh national strategy for maternal health 2019–2030	National nutrition policy 2015	Second national plan of action for nutrition 2016–2025	Addressing bottlenecks for the coverage of nutrition sensitive interventions in Bangladesh, BNNC 2021
Effectiveness	Effectively states the objectives of ensuring maternal and new-born health.	Effectively formulates the goal and objectives of ensuring optimal nutrition; however, lacks guidance to address overweight/obesity issues among women of reproductive age.	Effectively frames the goal and objectives of ensuring optimal nutrition for all	Effectively identifies overweight and obesity among women of reproductive age as a bottleneck.
Efficiency	Lacks clear guidance for efficiently allocating available resources and leveraging existing resources in an optimal way to address overweight/obesity issues among women of reproductive age.	Lacks any provision for allocating resources or leveraging existing resources in any way to address overweight/obesity issues among women of reproductive age.	Does not have any plan of action for efficiently allocating available resources and leveraging existing resources to address overweight/obesity issues among women of reproductive age.	This document successfully outlines the challenges that hinder the pathway of nutrition-sensitive interventions coverage quite efficiently.
Flexibility	Adaptability to keep pace with the changing scenario of health/nutrition challenges, knowledge, and gaps is insufficient.	Adaptability to keep pace with the changing scenario of health/nutrition challenges, knowledge, and gaps is non-existent.	Adaptability to keep pace with the changing scenario of health/nutrition challenges, knowledge, and gaps is inadequate.	Adaptability to keep pace with the changing scenario of health/nutrition challenges, knowledge, and gaps is sufficient.
Equity	Identifies overweight/obesity as a “persisting gap”; however, does not outline any activity for mothers and children.	Identifies overweight/obesity as a challenge but does not mention any roadmap of activities.	Does not specify any activities for non-pregnant and non-lactating women of reproductive age.	Covers almost all aspects of the reproductive life of a woman.

## 4 Discussion

### 4.1 Possible reasons for the increase in overweight and obesity among women age 15–49 years

A recent *Lancet* paper (10) showed that many developing countries were undergoing a similar case of DBM, a phenomenon known as “the new nutrition reality.” Although the economic revolution (39) has been fundamental for reduction of undernutrition, it has also fueled an expansion in overnutrition. Other significant reasons for this increase are spontaneous urban growth, migration to cities, income growth (40), and infrastructural advancements (41, 42).

Evidence shows that the rates of overweight/obesity are increasing faster in some regions of the world where certain micronutrient deficiencies are prevalent (43). This suggests that poor diets due to food and nutrition insecurity in countries like Bangladesh, resulting in micronutrient deficiencies, may contribute to the increase in overweight/obesity rates. For instance, the 2019–2020 Bangladesh National Micronutrient Survey revealed that 22% of the adult population in Bangladesh was suffering from vitamin D deficiency (44) and epidemiological studies (45) suggest that vitamin D deficiency, which is associated with impaired metabolism, results in more overweight and obesity.

In a cohort study in China (46), increases in overweight/obesity were substantially associated with decreased physical activity (47) due to technology (e.g., refrigerators, rice cookers, vacuum cleaners and washing machines) entering in workplaces and homes (48, 49) and improved transportation systems (50). Moreover, the surge in the number working women (51) has influenced the demand for ready-to-eat or ready-to-heat processed foods (52–54). Aggressive marketing of fast/junk foods and beverages, especially to children (55) are also growing faster than ever (56). Lastly, to meet the growing food consumption supply, commercial food production often using growth hormones, fertilizers, and genetically modified foods (57) have also been associated with the rise in weight gain and obesity (58–61).

### 4.2 Increased weight among women age 15–49 years: why is this a concern?

Out of the annual 3.4 million births in Bangladesh, about 0.8 million occur to overweight women and 0.5 million births occur to underweight women. If the current trend of DBM continues, pregnancies/births among overweight women is likely to increase rapidly.

The DBM has consequences for both maternal and child health. While underweight mothers have the risk of anemia, premature rupture of membranes, and antepartum/postpartum hemorrhage, overweight/obese mothers (62) has risk of developing perinatal complications (gestational diabetes, pregnancy related hypertension), and c-sections (63). Overweight/obese mothers also faces challenges with initiation and exclusive breastfeeding (64). In 2016, *The Lancet Diabetes and Endocrinology* published a series on maternal weight, highlighting the global burden of overweight/obesity among women and its potential serious implications for infant survival, growth, and development (65) and intergenerational consequences (Table 3) (66).

**Table 3:** Health risks for mothers and children due to maternal weight.

Health risks for mothers	Maternal underweight	Maternal overweight/ obese	Health risks for child	Maternal underweight	Maternal overweight/ obese
Infertility	–	✓	Perinatal mortality	✓	✓
Polycystic ovary syndrome	–	✓	Stillbirth	–	✓
Anemia	✓	–	Congenital anomalies	✓	✓
Gestational diabetes	–	✓	Low birth weight	✓	–
Gestational hypertension	–	✓	Fetal macrosomia	–	✓
Antepartum/Postpartum hemorrhage	✓	✓	Retarded fetal growth	✓	✓
Pre-eclampsia/Eclampsia	–	✓	Low APGAR score	–	✓
Premature rupture of membranes	✓	–	Difficulties in breastfeeding	–	✓
Cesarean delivery	✓	✓	Overweight/Obesity in later life	–	✓
Pre-term birth	–	✓			
Spontaneous miscarriage	–	✓			

Along with the identified health hazards for both the mother and child, DBM also creates a new challenge: the “dual-burden households” (67). This is the contradictory burden within households wherein one individual is overweight, and another is underweight (68). This challenge extremely complicates nutritional interventions (69) as interventions to reduce household level undernutrition often conflicts with overweight prevention programs and vice versa.

The Bangladesh *Adolescent Health and Wellbeing Survey 2019–20* (70) revealed that only 4% of ever-married adolescent females (15–19 years) were underweight compared with 16% being overweight. This shows that in Bangladesh the shift is taking place from an early age demanding relevant steps to be taken.

### 4.3 Reasons behind the gap in the national response

Figure 3 reveals that, as per the global BMI cut-off (BMI ≥25 kg/m^2^), Bangladesh experienced a shift in the dominant burden from underweight to overweight around 2012–2013. However, in the same figure, it is evident that until 2017–18, the shift was not very clear and noticeable, creating ambiguity on the actual state of malnutrition among women age 15–49 years.

After investigating the policy preparation process, it was found that although the Bangladesh National Strategy for Maternal Health timeline starts from 2019 and goes until 2030, the actual policy preparation process started around 2011. Moreover, this strategy was built on the 2001 Bangladesh National Strategy for Maternal Health, which focused mostly on strategies to reduce maternal mortality. In case of the National Nutrition Policy 2015, the policy preparation process started in early 2012 and was largely based on the information available at that time. A similar situation was observed for the Second National Plan of Action for Nutrition 2016–2025, which was prepared based on the 1997 National Plan of Action for Nutrition and the National Nutrition Policy 2015. In a nutshell, the preparatory activities for all three policies analyzed started before 2012–13. The shift from underweight and overweight took place around 2012–13. Thus, when the preliminary planning of these national responses happened, maternal undernutrition was predominant.

However, another relevant policy analysis (71) suggested that despite the government initiating several NCD-focused policies/programs, the absence of efficient planning, timely implementation, and effective monitoring; many of the activities has not sustained. A study (72) on NCD-risk factors among youths and national policy responses also stressed on the need for strengthening NCD-risk factors surveillance and launching effective SMART interventions targeting the youths. These two papers are backed by another recent paper (73), where it was mentioned that in Bangladesh, NCDs are somewhat prioritized in policy documents however, implementation remains weak. The government’s operational plan (OP) indicators mostly focus on the process and the readiness to provide NCD services at primary health care facilities are inadequate.

### 4.4 The way forward

As a nation, Bangladesh needs to recognize that DBM requires double-duty actions (74). In 2017, WHO published a policy brief (75), that acted as the forebear of the Global Nutrition Report 2022 (76), outlining the potential interventions for achieving double-duty actions and eliminating all forms malnutrition.

#### 4.4.1 The double-duty actions

As per the WHO, the double-duty actions include national responses—from policies to programs—that increase the likelihood of simultaneously reducing the burden of the two forms of malnutrition: undernutrition and overweight/obesity. Such actions have the ability to maximize resources and offer integrated solutions. Some Bangladesh-specific actions are:

#### 4.4.2 Gather evidence regularly and address DBM from early years

Young girls are more susceptible to both forms of malnutrition (77). Thus, implementing healthy dietary habits and physical exercise is important to address DBM. This can be done through school-based healthy food initiatives and by instituting community-based interventions to reach out-of-school (married/unmarried) and working young girls. Incorporation of social behavior change communication strategies in public health campaigns targeting youth with tailored messages to promote healthy eating habits and increased physical activity should be prioritized. Such approaches create the prospect of having a healthy and well-nourished population at the pre-conception stage.

#### 4.4.3 Targeted and segmented ANC programs

Guided nutrition counseling during ANC provides opportunities for having appropriate knowledge on optimal nutritional practices during pregnancy (78–80). Such counseling can steer women to have appropriate weight gain during pregnancy and provides protection against gestational diabetes/hypertension. This may result in ensuring safe delivery and prevent overweight/obesity later in life for the child. Achieving this would require a national guideline for promoting health dietary habits during pregnancy and advocate for optimum physical activity to mitigate the risk of obesity among both mothers and their offspring. Additionally, integration of nutritional education and counseling initiatives in existing maternal and child health programs can empower women to make informed choices and prevent obesity-related complications.

#### 4.4.4 Promote and protect appropriate IYCF practices

Such promotion and protection must incorporate practical interventions to ensure initiation of breastfeeding and exclusive breastfeeding (81, 82). Exclusive breastfeeding has the twofold advantages of catering ideal nutrition for the child and helping control postpartum weight gain. Proper complementary feeding also helps break the intergenerational cycle of the DBM.

#### 4.4.5 Effective regulation of aggressive marketing of junk and packaged foods and beverages

Aggressive marketing of junk and packaged foods and beverages influences children’s food preferences and when not done correctly can lead to childhood overweight/obesity. Effective regulation of aggressive marketing can facilitate prevention of overweight/obesity in early life and their impact on health at later stages (83). Focused and SMART national campaigns should be initiated for the reduction of high dependency on cereal-based foods and foods with high trans-fats, salt, and sugar and increased intake of animal-source foods.

#### 4.4.6 Provide healthy meals at schools

Providing healthy mid-day meals at schools (84, 85) has been found to be effective in increasing the availability and purchase of healthy foods and decreasing the purchase of unhealthy foods, with the potential to impact health. This would also provide an equitable opportunity for children coming from resource poor households in getting at least one healthy meal a day—somewhat addressing disparities in access to nutritious foods and promoting affordable healthy options.

#### 4.4.7 Develop a comprehensive food and nutrition security response framework

A comprehensive multisectoral food and nutrition security response framework followed by an action plan to address the double burden of malnutrition are essential to implement effective interventions. This would involve multiple stakeholders, including government agencies, non-governmental organizations, healthcare providers, and community leaders, in developing and implementing obesity prevention initiatives. Collaborative efforts aimed at creating supportive environments for healthy living and fostering community engagement are essential for sustainable outcomes.

## 5 Conclusion

The year 2023 provides an excellent opportunity to rectify the situation because Bangladesh is currently preparing for the next sector plan led by the MOHFW. Now is the time to adjust national policies addressing DBM among women age 15–49 years across pre-conception, pregnancy, and postnatal stages to ensure optimum maternal and child health.

## Data availability statement

Publicly available datasets were analyzed in this study. This data can be found at: https://dhsprogram.com/data/available-datasets.cfm.

## Ethics statement

Ethical approval was not required for the studies involving humans because ethical approval for this type of study is not required by our institutes. The data was analyzed from open-sourced data. Written informed consent to participate in these surveys were provided by the participants’ legal guardian/next of kin. The studies were conducted in accordance with the local legislation and institutional requirements.

## Author contributions

SK: Conceptualization, Writing – original draft, Methodology, Writing – review & editing. MH: Conceptualization, Formal analysis, Methodology, Project administration, Writing – original draft, Writing – review & editing. KJ: Conceptualization, Methodology, Writing – review & editing. KA: Writing – review & editing. SS: Writing – review & editing. AI: Writing – review & editing. GA: Supervision, Writing – review & editing.

## References

[1] World Health Organization. (2021). Malnutrition: key facts. Available at: https://www.who.int/news-room/fact-sheets/detail/malnutrition

[2] King FS Burgess A Quinn VJ Osei AK. Nutrition for developing countries. University of Oxford, UK: Oxford University Press (2015).

[3] Sekiyama M Jiang HW Gunawan B Dewanti L Honda R Shimizu-Furusawa H. Double burden of malnutrition in rural West Java: household-level analysis for father-child and mother-child pairs and the association with dietary intake. Nutrients. (2015) 7:8376–91. doi: 10.3390/nu7105399, PMID: 26445058 PMC4632419

[4] Ramirez-Zea M Kroker-Lobos MF Close-Fernandez R Kanter R. The double burden of malnutrition in indigenous and nonindigenous Guatemalan populations. Am J Clin Nutr. (2014) 100:1644S–51S. doi: 10.3945/ajcn.114.083857, PMID: 25411307

[5] Kimani-Murage EW Muthuri SK Oti SO Mutua MK Van De Vijver S Kyobutungi C. Evidence of a double burden of malnutrition in urban poor settings in Nairobi, Kenya. PLoS One. (2015) 10:e0129943. doi: 10.1371/journal.pone.0129943, PMID: 26098561 PMC4476587

[6] Kapoor S Anand K. Nutritional transition: A public health challenge in developing countries. London, United Kingdom: BMJ Publishing Group Ltd; (2002). p. 804–805, 56. 10.1136/jech.56.11.804 PMC1732057 12388563

[7] Organization WH. The double burden of malnutrition: Policy brief. Geneva, Switzerland: World Health Organization (2016).

[8] Food and Agriculture Organization of the United Nations (FAO), International Fund for Agricultural Development, World Food Programme. (2015). The State of Food Insecurity in the World 2015. *Meeting the 2015 international hunger targets: Taking stock of uneven Progress. Rome, Italy: FAO*.

[9] Collaboration NRF. Trends in adult body-mass index in 200 countries from 1975 to 2014: a pooled analysis of 1698 population-based measurement studies with 19.2 million participants. Lancet. (2016) 387:1377–96. doi: 10.1016/S0140-6736(16)30054-X 27115820 PMC7615134

[10] Popkin BM Corvalan C Grummer-Strawn LM. Dynamics of the double burden of malnutrition and the changing nutrition reality. Lancet. (2020) 395:65–74. doi: 10.1016/S0140-6736(19)32497-3, PMID: 31852602 PMC7179702

[11] Popkin BM. Nutrition in transition: the changing global nutrition challenge. Asia Pac J Clin Nutr. (2001) 10:S13–8. doi: 10.1046/j.1440-6047.2001.00211.x 11708577

[12] Iips I. National family health survey (NFHS-4), 2015–16. International Institute for Population Sciences (IIPS), Mumbai, India. (2017): 791–846.

[13] Gupta R Sharma K Gupta A Agrawal A Mohan I Gupta V. Persistent high prevalence of cardiovascular risk factors in the urban middle class in India: Jaipur heart Watch-5. J Assoc Physicians India. (2012) 60:11–6. 22799108

[14] Shukla H Gupta P Mehta H Hébert JR. Descriptive epidemiology of body mass index of an urban adult population in western India. J Epidemiol Community Health. (2002) 56:876–80. doi: 10.1136/jech.56.11.876 12388581 PMC1732045

[15] Hasan MM Ahmed S Soares Magalhaes RJ Fatima Y Biswas T Mamun AA. Double burden of malnutrition among women of reproductive age in 55 low-and middle-income countries: progress achieved and opportunities for meeting the global target. Eur J Clin Nutr. (2022) 76:277–87. doi: 10.1038/s41430-021-00945-y, PMID: 34040202 PMC8152189

[16] Khan SH Talukder SH. Nutrition transition in B angladesh: is the country ready for this double burden. Obes Rev. (2013) 14:126–33. doi: 10.1111/obr.12100, PMID: 24102686

[17] Rahman MA Halder HR Siddiquee T Farjana SA Roshid HO Khan B. Prevalence and determinants of double burden of malnutrition in Bangladesh: evidence from a nationwide cross-sectional survey. Forum Nutr. (2021) 46:1–12. doi: 10.1186/s41110-021-00140-w

[18] Hossain MI Rahman A Uddin MSG Zinia FA. Double burden of malnutrition among women of reproductive age in Bangladesh: a comparative study of classical and Bayesian logistic regression approach. Food Sci Nutr. (2023) 11:1785–96. doi: 10.1002/fsn3.3209, PMID: 37051361 PMC10084956

[19] Biswas RK Rahman N Khanam R Baqui AH Ahmed S. Double burden of underweight and overweight among women of reproductive age in Bangladesh. Public Health Nutr. (2019) 22:3163–74. doi: 10.1017/S1368980019002611, PMID: 31544733 PMC10260564

[20] Khan JR Gulshan J. Assessing the double burden of malnutrition among Bangladeshi reproductive-aged women: a comparison between unconditional and conditional quantile regression. Health Sci Reports. (2021) 4:e391. doi: 10.1002/hsr2.391, PMID: 34622024 PMC8485620

[21] Sarker AR Hossain Z Morton A. Drivers and distribution of the household-level double burden of malnutrition in Bangladesh: analysis of mother–child dyads from a national household survey. Public Health Nutr. (2022) 25:3158–71. doi: 10.1017/S1368980022002075, PMID: 36111605 PMC9991823

[22] Anik AI Rahman MM Rahman MM Tareque MI Khan MN Alam MM. Double burden of malnutrition at household level: a comparative study among Bangladesh, Nepal, Pakistan, and Myanmar. PLoS One. (2019) 14:e0221274. doi: 10.1371/journal.pone.0221274, PMID: 31419251 PMC6697370

[23] Tanwi TS Chakrabarty S Hasanuzzaman S. Double burden of malnutrition among ever-married women in Bangladesh: a pooled analysis. BMC Womens Health. (2019) 19:1–8. doi: 10.1186/s12905-019-0725-2 30704454 PMC6357418

[24] Black RE Victora CG Walker SP Bhutta ZA Christian P De Onis M. Maternal and child undernutrition and overweight in low-income and middle-income countries. Lancet. (2013) 382:427–51. doi: 10.1016/S0140-6736(13)60937-X 23746772

[25] Modjadji P Madiba S. Childhood undernutrition and its predictors in a rural health and demographic surveillance system site in South Africa. Int J Environ Res Public Health. (2019) 16:3021. doi: 10.3390/ijerph16173021, PMID: 31438531 PMC6747220

[26] Park D Lee J-H Han S. Underweight: another risk factor for cardiovascular disease?: a cross-sectional 2013 behavioral risk factor surveillance system (BRFSS) study of 491, 773 individuals in the USA. Medicine. (2017) 96:e8769. doi: 10.1097/MD.0000000000008769 29310352 PMC5728753

[27] Rutstein SO Rojas G. Guide to DHS statistics. Calverton, MD: ORC Macro. (2006) 38:78.

[28] National Institute of Population Research and Training-NIPORT/Bangladesh, Mitra and Associates/Bangladesh, ORC Macro. Bangladesh demographic and health survey 2004. Dhaka, Bangladesh: NIPORT, Mitra and Associates, and ORC Macro (2005).

[29] National Institute of Population Research and Training-NIPORT/Bangladesh, Mitra and Associates/Bangladesh, Macro International. Bangladesh demographic and health survey 2007. Dhaka, Bangladesh: NIPORT, Mitra and Associates, and Macro International (2009).

[30] National Institute of Population Research and Training-NIPORT/Bangladesh, Mitra and Associates, ICF International. Bangladesh demographic and health survey 2014. Dhaka, Bangladesh: NIPORT, Mitra and Associates, and ICF International (2016).

[31] National Institute of Population Research and Training (NIPORT), ICF. Bangladesh demographic and health survey 2017–18. Dhaka, Bangladesh, and Rockville, Maryland, USA: NIPORT and ICF (2020).

[32] DHS. Available datasets. (2020) Available from: https://dhsprogram.com/data/available-datasets.cfm.

[33] United Nations, Department of Economic and Social Affairs, Population Division. (2020). World Population Prospects 2022. Available at: https://population.un.org/wpp/

[34] Government of the People’s Republic of Bangladesh, Ministry of Health and Family Welfare (MOHFW). Bangladesh National Strategy for maternal health 2019–2030. Dhaka, Bangladesh: MOHFW (2019).

[35] Ministry of Health and Family Welfare (MOHFW). National Nutrition Policy 2015. Dhaka, Bangladesh: MOHFW (2015).

[36] Government of the People’s Republic of Bangladesh, Ministry of Health and Family Welfare (MOHFW). Second National Plan of action for nutrition (2016–2025). Dhaka, Bangladesh: MOHFW (2017).

[37] Bangladesh National Nutrition Council (BNNC). Addressing bottlenecks for the coverage of nutrition sensitive interventions in Bangladesh: Strategies and a conceptual model of community-targeted actions to overcome bottlenecks and improve coverage. Dhaka, Bangladesh: BNNC (2021).

[38] Centers for Disease Control and Prevention (CDC). CDC’s policy analytical framework. Atlanta, Georgia: Centers for Disease Control and Prevention, US Department of Health and Human Services (2013).

[39] Horton R Lo S. Nutrition: a quintessential sustainable development goal. Lancet. (2013) 382:371–2. doi: 10.1016/S0140-6736(13)61100-9, PMID: 23746782

[40] Bennett MK. The world's food. A study of the interrelations of world populations, national diets, and food potentials. Centers for Disease Control and Prevention, US Department of Health and Human Services (1954).

[41] Popkin BM. The shift in stages of the nutrition transition in the developing world differs from past experiences! Public Health Nutr. (2002) 5:205–14. doi: 10.1079/PHN2001295, PMID: 12027286

[42] Tschirley D Reardon T Dolislager M Snyder J. The rise of a middle class in east and southern Africa: implications for food system transformation. J Int Dev. (2015) 27:628–46. doi: 10.1002/jid.3107

[43] Monteiro CA Moura EC Conde WL Popkin BM. Socioeconomic status and obesity in adult populations of developing countries: a review. Bull World Health Organ. (2004) 82:940–6. PMID: 15654409 PMC2623095

[44] National Nutrition Services. National Micronutrient Survey, Bangladesh 2019–2020 – Preliminary findings. Dhaka, Bangladesh: National Nutrition Services (2022).

[45] Pereira M de Farias Costa PR Pereira EM de Lima Lago IR Oliveira AM. Does vitamin D deficiency increase the risk of obesity in adults and the elderly? A systematic review of prospective cohort studies. Public Health. (2021) 190:123–31. doi: 10.1016/j.puhe.2020.04.031 33453688

[46] Monda KL Adair LS Zhai F Popkin BM. Longitudinal relationships between occupational and domestic physical activity patterns and body weight in China. Eur J Clin Nutr. (2008) 62:1318–25. doi: 10.1038/sj.ejcn.1602849, PMID: 17637599

[47] Ng SW Popkin BM. Time use and physical activity: a shift away from movement across the globe. Obes Rev. (2012) 13:659–80. doi: 10.1111/j.1467-789X.2011.00982.x, PMID: 22694051 PMC3401184

[48] Monda KL Popkin BM. Cluster analysis methods help to clarify the activity—BMI relationship of Chinese youth. Obes Res. (2005) 13:1042–51. doi: 10.1038/oby.2005.122 15976147

[49] Bell AC Ge K Popkin BM. Weight gain and its predictors in Chinese adults. Int J Obes. (2001) 25:1079–86. doi: 10.1038/sj.ijo.0801651 11443510

[50] Bell AC Ge K Popkin BM. The road to obesity or the path to prevention: motorized transportation and obesity in China. Obes Res. (2002) 10:277–83. PMID: 11943837 10.1038/oby.2002.38

[51] Novta N Wong J. Women at work in Latin America and the Caribbean: International Monetary Fund Obesity Research (2017) 25:1. doi: 10.5089/9781475578928.001,

[52] Mincer J. Market prices, opportunity costs, and income effects. Measurement Econo. (1963) 25:67–82.

[53] Monteiro CA Levy RB Claro RM de Castro IRR Cannon G. Increasing consumption of ultra-processed foods and likely impact on human health: evidence from Brazil. Public Health Nutr. (2010) 14:5–13. doi: 10.1017/S1368980010003241 21211100

[54] Monteiro CA Moubarac JC Cannon G Ng SW Popkin B. Ultra-processed products are becoming dominant in the global food system. Obes Rev. (2013) 14:21–8. doi: 10.1111/obr.12107, PMID: 24102801

[55] Pries AM Filteau S Ferguson EL. Snack food and beverage consumption and young child nutrition in low-and middle-income countries: a systematic review. Matern Child Nutr. (2019) 15:e12729. doi: 10.1111/mcn.12729, PMID: 31225715 PMC6618154

[56] Huffman SL Piwoz EG Vosti SA Dewey KG. Babies, soft drinks and snacks: a concern in low-and middle-income countries? Matern Child Nutr. (2014) 10:562–74. doi: 10.1111/mcn.12126, PMID: 24847768 PMC4299489

[57] Rashad I. Obesity and diabetes: the roles that prices and policies play In: The economics of obesity, vol. 17: Emerald Group Publishing Limited (2006). 113–28. 19548550

[58] Reardon T Timmer CP Minten B. Supermarket revolution in Asia and emerging development strategies to include small farmers. Proc Natl Acad Sci. (2012) 109:12332–7. doi: 10.1073/pnas.1003160108, PMID: 21135250 PMC3412023

[59] Reardon T Berdegué J. The rapid rise of supermarkets in Latin America: challenges and opportunities for development. Wiley Online Library (2014).

[60] Reardon T Timmer CP Barrett CB Berdegué J. The rise of supermarkets in Africa, Asia, and Latin America. Am J Agric Econ. (2003) 85:1140–6. doi: 10.1111/j.0092-5853.2003.00520.x

[61] Reardon T Chen KZ Minten B Adriano L Dao TA Wang J. The quiet revolution in Asia's rice value chains. Ann N Y Acad Sci. (2014) 1331:106–18. doi: 10.1111/nyas.12391 24735399

[62] Grieger JA Hutchesson MJ Cooray SD Bahri Khomami M Zaman S Segan L. A review of maternal overweight and obesity and its impact on cardiometabolic outcomes during pregnancy and postpartum. Therapeutic Advan Reproductive health. (2021) 15:263349412098654. doi: 10.1177/2633494120986544 PMC7871058 33615227

[63] Chen C Xu X Yan Y. Estimated global overweight and obesity burden in pregnant women based on panel data model. PLoS One. (2018) 13:e0202183. doi: 10.1371/journal.pone.0202183 30092099 PMC6084991

[64] Amir LH Donath S. A systematic review of maternal obesity and breastfeeding intention, initiation and duration. BMC Pregnancy Childbirth. (2007) 7:1–14. 17608952 10.1186/1471-2393-7-9 PMC1937008

[65] Poston L Caleyachetty R Cnattingius S Corvalán C Uauy R Herring S. Preconceptional and maternal obesity: epidemiology and health consequences. Lancet Diabetes Endocrinol. (2016) 4:1025–36. doi: 10.1016/S2213-8587(16)30217-0, PMID: 27743975

[66] Kominiarek MA Rajan P. Nutrition recommendations in pregnancy and lactation. Med Clin. (2016) 100:1199–215. doi: 10.1016/j.mcna.2016.06.004, PMID: 27745590 PMC5104202

[67] Doak CM Adair LS Monteiro C Popkin BM. Overweight and underweight coexist within households in Brazil, China and Russia. J Nutr. (2000) 130:2965–71. doi: 10.1093/jn/130.12.2965, PMID: 11110855

[68] Doak CM Adair LS Bentley M Monteiro C Popkin BM. The dual burden household and the nutrition transition paradox. Int J Obes. (2005) 29:129–36. doi: 10.1038/sj.ijo.0802824, PMID: 15505634

[69] Jehn M Brewis A. Paradoxical malnutrition in mother–child pairs: untangling the phenomenon of over-and under-nutrition in underdeveloped economies. Econ Hum Biol. (2009) 7:28–35. doi: 10.1016/j.ehb.2009.01.007, PMID: 19246260

[70] National Institute of Population Research and Training (NIPORT), International Centre for Diarrhoeal Disease Research, Bangladesh (icddr,b), and Data for Impact. Bangladesh adolescent health and wellbeing survey 2019–20: final report. Dhaka, Bangladesh, and Chapel Hill, NC, USA: NIPORT, icddr,b, and Data for Impact (2021).

[71] Biswas T Pervin S Tanim MIA Niessen L Islam A. Bangladesh policy on prevention and control of non-communicable diseases: a policy analysis. BMC Public Health. (2017) 17:1–11. doi: 10.1186/s12889-017-4494-2 28629430 PMC5477154

[72] Biswas T Azzopardi P Anwar SN de Vries TD Encarnacion-Cruz LM Hasan MM. Assuring Bangladesh’s future: non-communicable disease risk factors among the adolescents and the existing policy responses. J Health Popul Nutr. (2022) 41:22. doi: 10.1186/s41043-022-00294-x, PMID: 35578321 PMC9109415

[73] Islam K Huque R Saif-Ur-Rahman K Kabir AE Hussain AE. Implementation status of non-communicable disease control program at primary health care level in Bangladesh: findings from a qualitative research. Public Health Prac. (2022) 3:100271. doi: 10.1016/j.puhip.2022.100271, PMID: 36101774 PMC9461504

[74] Hawkes C Demaio AR Branca F. Double-duty actions for ending malnutrition within a decade. Lancet Glob Health. (2017) 5:e745–6. doi: 10.1016/S2214-109X(17)30204-8 28528865

[75] World Health Organization (WHO). WHO double-duty actions: Policy brief. Geneva, Switzerland: WHO (2017).

[76] Global Nutrition Report. 2022 global nutrition report: Stronger commitments for greater action. Bristol, UK: Development Initiatives (2022).

[77] World Health Organization (WHO). Global nutrition policy review: What does it take to scale up nutrition action? Geneva, Switzerland: WHO (2013).

[78] World Health Organization (WHO). WHO recommendations on antenatal Care for a Positive Pregnancy Experience. Geneva, Switzerland: WHO (2016). 28079998

[79] United Nations Children’s Fund. UNICEF technical brief. Counselling to improve maternal nutrition. Considerations for programming with quality, equity and scale. New York: UNICEF, (2021). Available at: https://www.unicef.org/media/114566/file/Maternal%20Nutrition%20Counselling%20Brief.pdf(accessed March 23, 2023).

[80] Bhutta ZA Das JK Rizvi A Gaffey MF Walker N Horton S. Evidence-based interventions for improvement of maternal and child nutrition: what can be done and at what cost? Lancet. (2013) 382:452–77. doi: 10.1016/S0140-6736(13)60996-4, PMID: 23746776

[81] Armstrong J Reilly J. Breastfeeding and childhood obesity risk In: Obesity Research. US: NORTH AMER ASSOC STUDY OBESITY C/O DR MICHAEL JENSEN, MAYO MEDICAL CENTER (2001)

[82] Pearce J Langley-Evans S. The types of food introduced during complementary feeding and risk of childhood obesity: a systematic review. Int J Obes. (2013) 37:477–85. doi: 10.1038/ijo.2013.8, PMID: 23399778

[83] World Health Organization (WHO). Set of recommendations on the Marketing of Foods and non-Alcoholic Beverages to children. Geneva, Switzerland: WHO (2010).

[84] United Nations General Assembly. (2016). Seventieth session agenda item 15 (a/70/L.42). Available at: https://reliefweb.int/report/world/united-nations-decade-action-nutrition-2016-2025-a70l42

[85] Niebylski ML Lu T Campbell NR Arcand J Schermel A Hua D. Healthy food procurement policies and their impact. Int J Environ Res Public Health. (2014) 11:2608–27. doi: 10.3390/ijerph110302608, PMID: 24595213 PMC3986994

[86] Appropriate body-mass index for Asian populations and its implications for policy and intervention strategies. The Lancet. 363:157–163., PMID: 14726171 10.1016/S0140-6736(03)15268-3

