# Supplementary material for: Changing paradigm of malnutrition among Bangladeshi women of reproductive age and gaps in national Nutrition Policies and Action Plans to tackle the emerging challenge
Source: Front Public Health. 2024 Oct 16;12:1341418. doi: 10.3389/fpubh.2024.1341418 (PMC11524151; doi:10.3389/fpubh.2024.1341418)
Supplement: Supplementary file 1 [file Table_1.DOCX]

Supplementary Material

**The changing paradigm of malnutrition among Bangladeshi women of reproductive age: analysis of national data and systematic review of policies to reassess national policy, planning, and programmatic responses.**

Shusmita Khan ^1,2^*, M Moinuddin Haider ^3^, Kanta Jamil^6^, Karar Zunaid Ahsan^2^, Saiqa Siraj^5^_,_ Afrin Iqbal^4^, Gustavo Angeles^2^

**Correspondence***

Shusmita Khan

[shusmita@email.unc.edu](mailto:shusmita@email.unc.edu)

**Supplementary Tables**

**Table S1.** Sample characteristics, by survey

|  | **BDHS**  **2004** | **BDHS**  **2007** | **BDHS**  **2011** | **BDHS**  **2014** | **BDHS**  **2017-18** |
| --- | --- | --- | --- | --- | --- |
| Women from whom anthropometric data were collected | Ever married; ages 10–49 | Ever married; ages 15–49 | Ever married; ages 15–49 | Ever married; ages 15–49 | Ever married; ages 15–49 |
| Number of women from whom anthropometric data were collected | 11,440 | 10,996 | 17,842 | 17,863 | 20,127 |
| Number of women who fulfilled the following conditions:  (a) 15–49 years  (b) not pregnant  (c) did not give birth 2 months before the survey  (d) had both height and weight data to calculate BMI  (Others were excluded from the analysis.) | 10,334 | 9,997 | 16,023 | 16,451 | 18,411 |
| Note: CMC dates were applied for examining condition (c). For this analysis, the BDHS 2017–18 used exact dates in the tabulation of the published report and got a total of 18,328 women (weighted number). To be consistent with the BDHS 2004, 2007, and 2011 that used CMC dates, we have applied CMC dates for 2017–18 as well. | | | | | |

**Table S2.** Estimated number of ever married women of reproductive age (EMWRA), overweight/obese EMWRA, and underweight EMWRA, by BDHS years

| **Row** | **Indicators** | **2017-18 (BDHS year)** |
| --- | --- | --- |
| R1 | Total population in Bangladesh (million)^1^ | 159.7 |
| R2 | Proportion of EMWRA in total population^2^ | 0.2394 |
| R3 | Proportion of underweight among EMWRA^2^ | 0.1190 |
| R4 | Proportion of overweight among EMWRA^2^ | 0.3240 |
| R5 | Total EMWRA in the population (million) **[R1 x R2]** | 38.2 |
| R6 | Total underweight EMWRA (million) **[R3 x R5]** | 4.5 |
| R7 | Total overweight EMWRA (million) **[R4 x R5]** | 12.4 |
| ^1^ Source: World Population Prospects 2019 <https://population.un.org/wpp/Download/Files/1_Indicators%20(Standard)/EXCEL_FILES/1_Population/WPP2019_POP_F01_1_TOTAL_POPULATION_BOTH_SEXES.xlsx>  ^2^ Sources: BDHS 2004, 2007, 2011, 2014, and 2017–18 <https://dhsprogram.com/>  Note: Overweight includes overweight and obese women. | | |

**Table S3.** Estimates related to nutritional status of EMWRA and children born to the EMWRA, BDHS 2017–18

| **Row** | **Indicators (totals are in million)** | **2017-18 (BDHS year)** |
| --- | --- | --- |
|  |  |  |
| R1 | Total EMWRA in the population ^1^ | 38.2 |
| R2 | Proportion of EMWRA who give birth in a year ^2^ | 0.0870 |
| R3 | Mean children born to each EMWRA who gave birth in last year ^2^ | 1.01 |
| R4 | Total children born in a year to EMWRA **[R1 x R2 x R3]** | 3.4 |
| R5 | Proportion of children born to EMWRA 15–19 ^2^ | 0.2330 |
| R6 | Proportion of children born to EMWRA 20–29 ^2^ | 0.5864 |
| R7 | Proportion of children born to EMWRA 30–39 ^2^ | 0.1736 |
| R8 | Proportion of children born to EMWRA 40–49 ^2^ | 0.0070 |
|  |  |  |
| R9 | Proportion of children born in a year to 15–19 EMWRA ^2^ | 0.2330 |
| R10 | Proportion of 15–19 EMWRA who are underweight ^2^ | 0.2430 |
| R11 | Proportion of 15–19 EMWRA who are overweight ^3^ | 0.1160 |
| R12 | Total children born to 15–19 EMWRA **[R4 x R9]** | 0.7842 |
| R13 | Total children born to 15–19 underweight EMWRA **[R12 x R10]** | 0.1906 |
| R14 | Total children born to 15–19 overweight EMWRA **[R12 x R11]** | 0.0910 |
|  |  |  |
| R15 | Proportion of children born in a year to 20–29 EMWRA ^2^ | 0.5864 |
| R16 | Proportion of 20–29 EMWRA who are underweight ^2^ | 0.1240 |
| R17 | Proportion of 20–29 EMWRA who are overweight ^3^ | 0.2740 |
| R18 | Total children born to 20–29 EMWRA **[R4 x R15]** | 1.9737 |
| R19 | Total children born to 20–29 underweight EMWRA **[R18 x R16]** | 0.2447 |
| R20 | Total children born to 20–29 overweight EMWRA **[R18 x R17]** | 0.5408 |
|  |  |  |
| R21 | Proportion of children born in a year to 30–39 EMWRA ^2^ | 0.1736 |
| R22 | Proportion of 30–39 EMWRA who are underweight ^3^ | 0.0880 |
| R23 | Proportion of 30–39 EMWRA who are overweight ^3^ | 0.3910 |
| R24 | Total children born to 30–39 EMWRA **[R4 x R21]** | 0.5843 |
| R25 | Total children born to 30–39 underweight EMWRA **[R24 x R22]** | 0.0514 |
| R26 | Total children born to 30–39 overweight EMWRA **[R24 x R23]** | 0.2285 |
|  |  |  |
| R27 | Proportion of children born in a year to 40–49 EMWRA ^2^ | 0.0070 |
| R28 | Proportion of 40–49 EMWRA who are underweight ^3^ | 0.1080 |
| R29 | Proportion of 40–49 EMWRA who are overweight ^3^ | 0.3780 |
| R30 | Total children born to 40–49 EMWRA **[R4 x R27]** | 0.0236 |
| R31 | Total children born to 40–49 underweight EMWRA **[R30 x R28]** | 0.0025 |
| R32 | Total children born to 40–49 overweight EMWRA **[R30 x R29]** | 0.0089 |
|  |  |  |
| R33 | Total children born to underweight EMWRA **[R13 x R19 x R25 x R31]** | 0.5234 |
| R34 | Total children born to overweight EMWRA **[R14 x R20 x R26 x R32]** | 0.8318 |
| R35 | Total children born to underweight and overweight EMWRA **[R33 x R34]** | 1.3552 |
| ^1^ Table S2; ^2^ Calculated from BDHS data; ^3^ BDHS 2004, 2007, 2011, 2014, and 2017–18 reports | | |

**Table S4.** Framing questions for analysis of policies

| **Framing questions** | |
| --- | --- |
| - What is the policy lever—is it legislative, administrative, regulatory, other? - What level of government or institution will implement this policy? - How does the policy work/operate? (e.g., is it mandatory? will enforcement be necessary?) - How is it funded? - Who is responsible for administering the policy? - What are the objectives of the policy? - Who are the target populations of the policy? - What is the legal landscape surrounding the policy (e.g., court rulings, constitutionality)? - What is the historical context (e.g., has the policy been developed based on an updated country context analysis and needs assessment)? - What is the value-added point of the policy? - What are the expected short, intermediate, and long-term outcomes? - What might be the unintended positive and negative consequences of the policy? | |
| **Criteria** | **Questions** |
| **Formulation stage** | 1. How does the policy address the problem? 2. What is the magnitude, reach, and distribution of benefit and burden? 3. What population will benefit? How much? When? 4. Will the policy impact health disparities/health equity? How? 5. Are the data updated based on which the policy is being formulated? 6. Are there gaps in the data/evidence-base? |
| **Followed up by a strategy** | 1. Has the policy been followed up by a strategy? 2. Is the strategy country specific and culturally appropriate? |
| **Followed up by a plan of action** | 1. Has the strategy been followed up by an itemized plan of action? 2. Are the activities costed and budgeted for? 3. Have the responsible bodies been identified? 4. Has a definite timeline for each action item been identified? |
| **M&E** | 1. Are there indicators to assess the effectiveness of the policy points/activities 2. Are the policy/activity items flexible for revision? 3. Do the policy/activity items impact health disparities/health equity? 4. What are the institutional constraints? 5. Are each of the activities costed/budgeted? 6. Has the community accepted the activities? |
| **Policy revision** | 1. Is there any provision for policy revision? 2. Who will revise the policy? 3. Has there been any timeline fixed for assessment to determine if revision is required? |

**Table S5.** Scoring process to determine policy effectiveness

| **Criteria** | **Questions** | **Scoring** |
| --- | --- | --- |
| **Formulation stage** | 1. How does the policy address the problem? | ❏ Low ❏ Medium ❏ High ❏ Not applicable ❏ Not available |
|  | 1. What is the magnitude, reach, and distribution of benefit and burden? | ❏ Low ❏ Medium ❏ High ❏ Not applicable ❏ Not available |
|  | 1. What population will benefit? How much? When? | ❏ Low ❏ Medium ❏ High ❏ Not applicable ❏ Not available |
|  | 1. Will the policy impact health disparities/health equity? How? | ❏ Low ❏ Medium ❏ High ❏ Not applicable ❏ Not available |
|  | 1. Are the data updated based on which the policy is being formulated? | ❏ Low ❏ Medium ❏ High ❏ Not applicable ❏ Not available |
|  | 1. Are there gaps in the data/evidence-base? | ❏ Low ❏ Medium ❏ High ❏ Not applicable ❏ Not available |
| **Followed up by a strategy** | 1. Has the policy been followed up by a strategy? | ❏ Low ❏ Medium ❏ High ❏ Not applicable ❏ Not available |
|  | 1. Is the strategy country specific and culturally appropriate? | ❏ Low ❏ Medium ❏ High ❏ Not applicable ❏ Not available |
| **Followed up by a plan of action** | 1. Has the strategy been followed up by an itemized plan of action? | ❏ Low ❏ Medium ❏ High ❏ Not applicable ❏ Not available |
|  | 1. Are the activities costed and budgeted for? | ❏ Low ❏ Medium ❏ High ❏ Not applicable ❏ Not available |
|  | 1. Have the responsible bodies been identified? | ❏ Low ❏ Medium ❏ High ❏ Not applicable ❏ Not available |
|  | 1. Has a definite timeline for each action item been identified? | ❏ Low ❏ Medium ❏ High ❏ Not applicable ❏ Not available |
| **M&E** | 1. Are there indicators to assess the effectiveness of the policy points/activities? | ❏ Low ❏ Medium ❏ High ❏ Not applicable ❏ Not available |
|  | 1. Are the policy/activity items flexible for revision? | ❏ Low ❏ Medium ❏ High ❏ Not applicable ❏ Not available |
|  | 1. Do the policy/activity items impact health disparities/health equity? | ❏ Low ❏ Medium ❏ High ❏ Not applicable ❏ Not available |
|  | 1. What are the institutional constraints? | ❏ Low ❏ Medium ❏ High ❏ Not applicable ❏ Not available |
|  | 1. Are each of the activities costed/budgeted? | ❏ Low ❏ Medium ❏ High ❏ Not applicable ❏ Not available |
|  | 1. Has the community accepted the activities? | ❏ Low ❏ Medium ❏ High ❏ Not applicable ❏ Not available |
| **Policy revision** | 1. Is there any provision for policy revision? | ❏ Low ❏ Medium ❏ High ❏ Not applicable ❏ Not available |
|  | 1. Who will revise the policy? | ❏ Low ❏ Medium ❏ High ❏ Not applicable ❏ Not available |
|  | 1. Has there been any timeline fixed for assessment to determine if revision is required? | ❏ Low ❏ Medium ❏ High ❏ Not applicable ❏ Not available |
